# Needle consideration in umbilical two-port laparoscopic percutaneous extraperitoneal closure for patent processus vaginalis of children: hook-needle or forcep-needle

**DOI:** 10.1186/s12893-022-01866-8

**Published:** 2022-12-02

**Authors:** Yuanhong Xiao, Jing Zhang

**Affiliations:** 1 Department of Pediatric Surgery, The Seventh Medical Center, Chinese PLA General Hospital, Nan Men Cang Hutong 5th, Dong Si Shi Tiao, Dong Cheng District, Beijing, 100700 China; 2 Department of Pediatric Surgery, Beijing United Family Hospital and Clinics, 2 Jiangtai Road, Chaoyang District, Beijing, 100015 China

**Keywords:** Boys, Child, Laparoscopy, Minimal invasive surgery, Patent processus vaginalis closure

## Abstract

**Background:**

Although umbilical two-port laparoscopic percutaneous extraperitoneal closure for the treatment of processus vaginalis patency of children has been verified to be safe and effective, the improvements of technical skills and instruments have been always on their ways. Recently, forcep-needle has begun to be used. In this study, we compared forcep-needle with hook-needle in this minimal invasive procedure for children suffered from hernia or hydrocele, with the aim to evaluate the instrumental convenience of the two needles.

**Methods:**

From July 2021 to May 2022, we begun to use hook-needle or forcep-needle in umbilical two-port laparoscopic percutaneous extraperitoneal closure for children suffered from hernia or hydrocele. The hook-needle group included nineteen children and the forcep-needle group included twenty-four ones. The data of the patients age, sex, side, operation time, postoperative hospital-stay, follow-up time, postoperative complications were evaluated. Common silk thread was used to encircle the internal ring preperitoneally.

**Results:**

There were no statistical differences between the two groups for the following items: age, sex, side, operation time, postoperative hospital-stay and postoperative complications (P > 0.05). The follow-up time of the hook-needle group was longer than that of the forcep-one (P = 0.0020). No open transfer happened for all the patients. One hydrocele boy in the hook-needle group reoccurred 1 month postoperatively due to the peritoneal broken. The single pole retreating of the hook-needle accompanied with chaotic movements, while for that of the forcep-needle, the double-arm retreating movements were more orderly. The outer surface of the forcep-needle was smooth without restrain, as for the hook-needle, an inlaid barb held the danger of brokening the peritoneum.

**Conclusion:**

In our preliminary experience of umbilical two-port laparoscopic percutaneous extraperitoneal closure using a hook-needle or a forcep-needle, in view of the instrumental convenience and safety, the double-arm and smooth outer surface designs of the forcep-needle contained more spatial orientation perceptions and safety.

## Background

Since 2006, when Takahara H firstly reported their innovative method of laparoscopic percutaneous extraperitoneal closure (LPEC) for children who suffered from hernia [1, 2], surgeons have begun to study and practise this novel minimally invasive procedure for pediatric patients with hernia or hydrocele [3–11]. Although the effectiveness and safety of this procedure have been verified, improvements of technical skills and instruments have always been on their ways [12–20]. Commonly, laparoscopic ergonomics were often concerned with the instrumental convenience and patients’ safety [21, 22].

In our previous research of single-port umbilical LPEC for girls with an epidural needle [23], when piercing the needle, we used the towel forceps to pull the abdominal wall to enhance the spatial orientation perception and eye-hand coordination. Similarly, in the practice of umbilical single-site two-port LPEC for children, in order to conquer the instrumental collisions in the limited umbilical space, we actioned the two poles synchronously parallelly and sagittally to form a solo-like surgery fashion [24]. A central holed needle could accomplish the internal ring being encircled by a common silk thread.

Recently, in our umbilical two-port LPEC procedures for children suffered from hernia or hydrocele, we began to use a novel forcep-needle which has not been discussed in the literatures.

## Methods

### Population and data collection

**Fig. 1:**
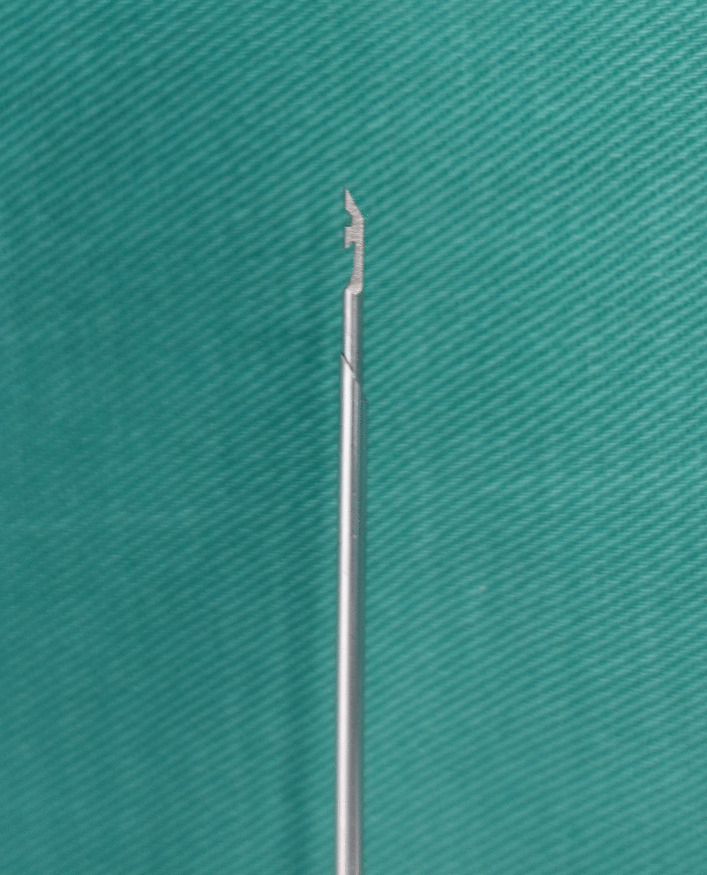
The hook-needle with an inlaid barb

**Fig. 2:**
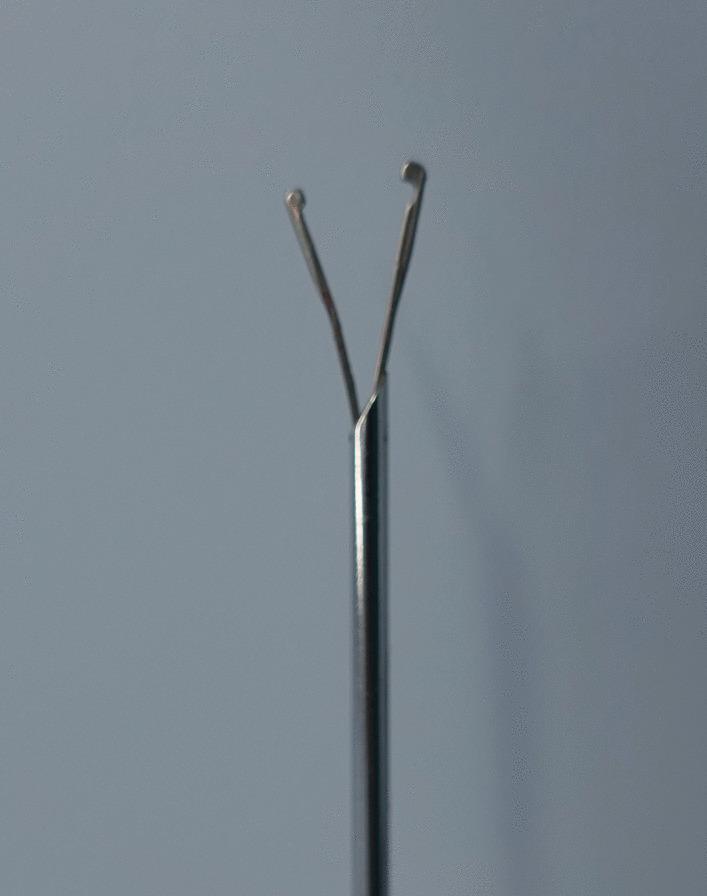
The forcep-needle with double-arm, a pair of inward distal jaws and a smooth outer surface

From July 2021, the authors began to use hook-needle (Fig. 1) or forcep-needle (Fig. 2) to complete umbilical two-port laparoscopic percutaneous extraperitoneal closure for children who suffered from hydrocele or hernia. The patient data of age, sex, affected side, operation time, postoperative hospital stay, follow-up time, complications were evaluated (Tables 1 and 2). No open transfers occurred for all the patients. One hydrocele boy in the hook-needle group reoccured 1 month postoperatively. No complications occurred in the forcep-needle group. The parents signed the informed contents for their children’s laparoscopic surgeries. All the parents signed the informed contents to participate in this research. We confirmed that the surgical procedures were in accordance with the relevant guidelines and regulations.

**Table 1:** Patient characteristics

Items	Hook-needle group	Forcep-needle group	P value
Cases	19	24	
Age (years)	4.579 ± 2.201 (1.250–8.000)	4.455 ± 2.457 (1.000–10.000)	0.8626
Sex			
M	15	23	0.2163
F	4	1	
Sides	34	40	
Unilateral	4	8	
Bilateral	15	16	0.5828

**Table 2:** Patient outcomes

Items	Hook-needle group	Forcep-needle group	P value
Operation time (min)	37.053 ± 9.975 (23.000–55.000)	36.792 ± 11.721 (9.000–69.000)	0.9376
Postoperative saty (days)	1.474 ± 0.612 (1–3)	2.042 ± 1.268 (1–7)	0.0618
Follow-up time (months)	8.421 ± 2.652 (3.000–11.000)	5.500 ± 3.148 (1.000–10.000)	0.0020
Reoccurence (case)	1	0	0.4419

### Surgical processes

The patient was supine under a tracheal intubation general anesthesia. The operator sited at the left side of the patient and the assistant at the right. The video screen was sited at the patient feet side. Two 5 mm incisions were made at the upper right and lower left of the umbilical verge respectively for accommodations of the camera (5 mm, 30° optics) and the disposable pre-bent auxiliary forceps. The pneumoperitoneum pressure of carbon dioxide was 8–12 mmHg. Firstly, camera inspections were made to preclude the accidental injuries of the vessels or viscera. Then further confirmations of the affected side and the opposite asymptomatic processus vaginalis patency were made. All the processus vaginalis patencies would be closed.

A 2 mm inguinal incision was made just above the internal ring level. A silk thread was folded back into double strands to form a U-type. The folded middle portion of the thread was held by the needle, and the two-strand ends of the thread maintained outside of the abdominal wall. First, the needle pierced into the preperitoneal space of the internal ring. With the auxiliary forceps assistance, the needle dived along the internal ring to seperate the spermatic vessels and vas away from the peritoneum. Then, the needle broke the peritoneum into the cavity. After the loaded thread loop was left in the cavity, the needle retraced to the original piercing site subcutaneously. Next, the needle dived along the residual preperitoneal space of the internal ring. Once arriving at the peritoneum piercing site, through which the needle came into the cavity again. With the auxiliary forceps help, the thread loop was captured by the hook or the forceps. Once the thread loop had been fastened, the needle retraced preperitoneally to the abdominal wall. So far, the internal ring had been completely encircled by the thread. Then, a knot-tie outside of the abdominal wall was made to close the internal ring eventually. The tie was buried subcutaneously. The incisions were sutured with absorbable thread subcutaneously, and then covered with tissue glue. The scrotal liquid was sucked out with a syringe needle if existent. Waterproof dressings covered the incisions. During the whole procedure, we cared not to damage the inferior epigastric, femoral and external iliac vessels.

### Data analysis

CHISS (Chinese High Intellectualized Statistical Software) software version 2004 was used for data analysis. T-test was used for quantitative data analysis. Pearson Chi-square was used for qualitative data analysis. P < 0.05 was defined as a statistical significance.

## Results

For the hook-needle group and forcep-needle group, the data of the patient age, sex and side had no statistical significances (P = 0.8626, 0.2163, 0.5828, respectively). The operative time and postoperative stay in hospital of the two groups had no statistical significance (P = 0.9376, 0.0618, respectively). The follow-up time of the hook-needle group was longer than that of the forcep-needle group (P = 0.0020). One hydrocele boy in the hook-needle group reoccured one month postoperatively. No other postoperative complications and transfers occurred. The statistical significance of the complication between the two groups was 0.4419 (Table 2).

## Discussion

The hook-needle or forcep-needle were all constituted of an outer metal sheath and an inner core. The sheath tip was arc-shaped for puncture. And the core could be pushed out of or withdrawn into the sheath freely with one hand. The hook-needle core was sculptured into a barb (Fig. 1), which exposed in the cavity for thread loading and capturing. So the outer surface of the hook-needle was not smooth. While, for that of the forcep-needle, the pole was made up of two arms which had jaws at their distal portions (Fig. 2). The jaws were inwardly facing. When closed, they bited compactly and the outer surface was smooth. The designs of the single pole or double-arm of the needles implied different spatial orientation perceptions. In this study, we perceived more chaotic retreating movements of the hook-needle during its single pole withdrawing into the sheath. Namely, the retreating path could not be kept in a steady straight line. Vahe Karimyan had pointed out this defective straight line movements were mainly caused by the defective spatial orientation perceptions of the laparoscopic two-dimensional sight. And vice versa, these chaotic collisional retreating movements worsened the poor spatial awareness further [25]. And in terms of mathematical and natural points of view proposed by C S Royden [26], human beings might have the curve line illusion of a real straight line one. To overcome this visual localization coordination defects, human applied active, rapid saccades of the eyes to re-establish mapping with a very limited effectiveness [27, 28]. The laparoscopic haptic feedback functions had been verified by researchers [29, 30]. Vasileios Lahanas had suggested that the grasping action which accompanied by a force feedback producing, could be of crucial importance for technically demanding tasks [29]. We found that for the forcep-needle, with the two arms being opened, the consequent counter-acting forces transmitted to the hand were perceived by the user. These counter-acting forces produced by the instrument were beneficial for the spatial orientation perceptions for the user. The opening two arms of the forcep-needle formed a triangular plane, on which the core could be retreating stably along a straight line. As for that of the hook-needle, the single pole retreated in an unfamiliar stereoscopic space without any specific reference frames. To overcome spatial perception scarceness, more touch with adjacent tissues occurred to obtain tactile sensation. This chaotic contact of the barb with surrounding tissues made the peritoneum in a danger of being broken. The hydrocele reoccrence of this study was attributed to the chaotic retreating movements of the inlaid hook.

**Fig. 3:**
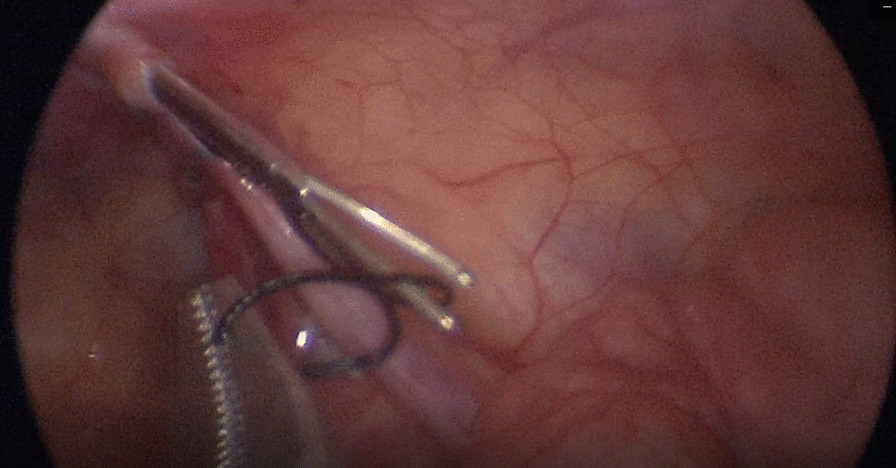
The thread capture by the forceps was closed after the jaws bited

**Fig. 4:**
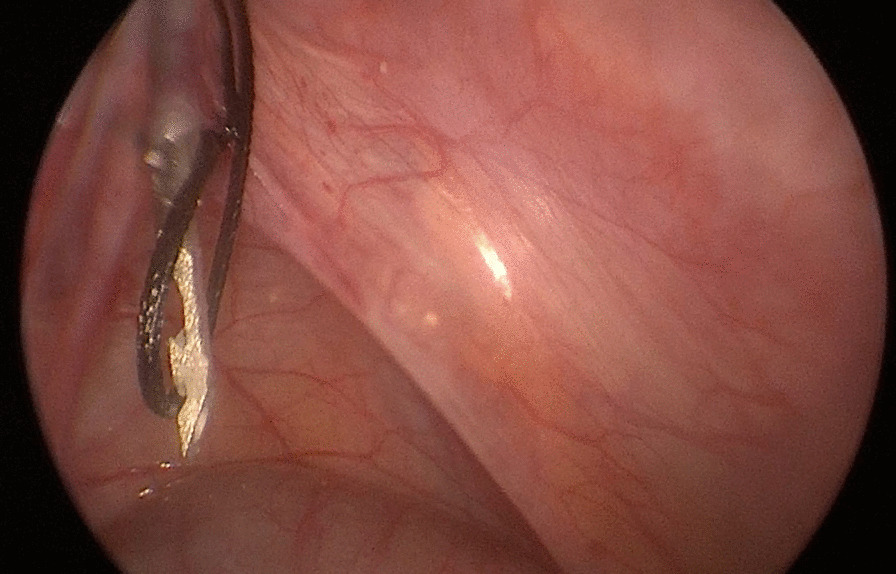
The thread capture by the hook was not fastened and had the possibility of escaping

In addition, it was more fastened when the thread loop clamped by the forceps (Fig. 3) than hooked by the barb (Fig. 4). For the former was a closed capture and the latter was an opened one.

## Conclusions

In our preliminary experience of umbilical two-port laparoscopic percutaneous extraperitoneal closure using a hook-needle or a forcep-needle, in view of the instrumental convenience and safety, the double-arm and smooth outer surface designs of the forcep-needle contained more spatial orientation perceptions and safety.

## Data Availability

All data is contained within the manuscript. The datasets used and analysed during the current study available from the corresponding author on reasonable request.

## Abbreviations

CHISS: Chinese High Intellectualized Statistical Software
LPEC: Laparoscopic percutaneous extraperitoneal closure
